# Serum level of semicarbazide-sensitive amine oxidase in children with ADHD

**DOI:** 10.1186/1744-9081-2-5

**Published:** 2006-01-27

**Authors:** Veit Roessner, Henrik Uebel, Andreas Becker, Georg Beck, Stefan Bleich, Aribert Rothenberger

**Affiliations:** 1 Department of Child and Adolescent Psychiatry, University of Goettingen, Von-Siebold-Str. 5, D-37075 Goettingen, Germany; 2 Department of Psychiatry and Psychotherapy, University of Erlangen, Schwabachanlage 6, D-91054 Erlangen, Germany

## Abstract

**Background:**

The objective of this study was to analyze the extracellularly acting semicarbazide-sensitive amine oxidase (SSAO) serum levels in children with ADHD for the first time. SSAO is known to show deviations from normal in various somatic disorders and to interplay with the intracellularly active MAO. In humans two forms of SSAO a circulating form in plasma and a membrane-bound form are involved in monoaminergic metabolism.

**Methods:**

We analyzed serum levels of SSAO in 27 children meeting ICD-10 criteria of Hyperkinetic Disorder (F90) or DSM-IV criteria of ADHD combined type by HPLC method and fluorimetric detection. A group of 42 healthy volunteers within the same age range (7.0 – 14.0 years) served as controls.

**Results:**

No significant differences between children with ADHD (SSAO activity M = 773, SD = 217 mU/l) and healthy controls (SSAO activity M = 775, SD = 256 mU/l) in SSAO serum levels were found (F = 2.18; p > 0.14). Further, stimulant medication status had no influence on the result (F = 2.52; p > 0.11).

**Conclusion:**

There is no evidence for a deviation of SSAO serum activity in ADHD. Hence, extracellularly acting SSAO does not seem to be a promising factor for further research in ADHD. But progress in knowledge of its physiologic role and of the relationship between the membrane-bound and the circulating serum form may open new avenues for research on SSAO in ADHD.

## Background

There is increasing evidence that deviations of the dopaminergic, noradrenergic, and serotonergic systems are involved in attention deficit hyperactivity disorder (ADHD) [1]. Several biogenic amines of these systems are degraded by oxidative deamination via the intracellular enzyme group monoamine oxidases (MAO). Thus the latter play an important role concerning the modification of signal transduction within these neurotransmitter systems [2].

In children with ADHD, significantly lower levels of platelet MAO activity seem to be associated with increased impulsivity and inattention [3]. In addition, different medications with MAO inhibiting properties have been shown to be effective in the pharmacological treatment of ADHD (for a review see [4,5]). Further, MAO seems to be a good candidate for genetic investigations of ADHD since DNA variations in this gene may play a role in the predisposition to the disorder. However, studies showed mixed results [6-9] and focusing on the intracellularly acting MAO only seems to be a too narrow look.

Other aminoxidases like the semicarbacide sensitive amino oxidase (SSAO) may also be involved in ADHD. Since SSAO acts extracellularly, it may work complementary to the intracellular activity of MAO [10]. Additionally, there is direct physiological interplay between the activities of SSAO and MAO [11].

In humans two forms of SSAO have been detected: a circulating form in plasma and a membrane-bound form. Both forms of SSAO metabolize several aromatic and aliphatic primary amines including dopamine while generating at the same time hydrogen peroxide and ammonia. The presence of SSAO in human cerebrovascular tissues and in endothelial cells from microvessels (including the blood-brain barrier) [12,13] (for a review see[14]) supports its impact on CNS metabolism and makes it a candidate to be investigated in ADHD.

Therefore, to shed more light on this issue our pilot-study analyzed the extracellularly acting SSAO serum levels in ADHD for the first time.

## Methods and materials

### Patients

A total of 27 children meeting ICD-10 criteria of Hyperkinetic Disorder (F90) or DSM-IV criteria of ADHD combined type (assessment by clinical interview, physical examination and Diagnostic Checklist, DCL-HKS [15]) (age range 7.0 – 14.0 years) were included. All were recruited sequentially from the outpatient clinic of the Department of Child and Adolescent Psychiatry of the University of Erlangen-Nuremberg (n = 12) and the University of Goettingen (n = 15). Further inclusion criteria were the following: intelligence IQ ≥ 85, body weight > 20 kg. Exclusion criteria were a clinical diagnosis of a developmental disorder or psychosis, depressive disorder, anxiety disorder, tic disorder, substance abuse, previous or recent epilepsy, EEG registration indicating epileptiform activity, previous/recent internal medicine or other neurological problems or treatment with known SSAO-inhibitory substances (i.e. tricyclic antidepressants) or other antidepressants. 13 children received methylphenidate, 14 were medication free.

A group of 42 healthy volunteers within the same age range (7.0 – 14.0 years) served as controls.

In view of the fact that SSAO circulating in plasma is quite stable [16] only one blood sample was collected from each participant. Because in SSAO activity no sex differences and no effect of body position (standing, sitting, supine) as well as of time of day of blood sampling has been observed[17,18], we did not control for these conditions.

This pilot study was carried out with the approval of the local Ethics Committees of the Medical Faculties of the Universities of Erlangen-Nuremberg and Goettingen. Written informed consent has been obtained from all parents and the patients able to write; younger children gave their oral assent.

### Laboratory methods

The SSAO activity was determined according to a method of van Dijk et al. [19], modified in a few points. Initially, the enzymes MAO-A and MAO-B were inhibited in a 30-minute pre-incubation with chlorgyline, followed by conversion of benzylamine to benzaldehyde catalyzed by SSAO at 37°C over 60 minutes.

After precipitation of the proteins contained in the reaction mixture and stopping of the reaction, the benzaldehyde formed was derivatized with 5,5-dimethyl-1,3-cyclohexandione (dimedone) from the supernatant, which specifically reacts with aldehydes. The derived material was quantified using an isocratic HPLC method and fluorimetric detection (λ excitation 386 nm, λ emission 451 nm). For evaluation of the chromatograms, the software ChromGate, Version 2.8 (Knauer) was used.

In the present study, in contrast to the method of van Dijk et al. [19], serum was used as sample material instead of plasma. In addition, semicarbazide was used as an inhibitor for the sample blanks, since the MDL-72,145A used by van Dijk et al. [19] does not have any particular advantages over semicarbazide and the inhibition of the SSAO with semicarbazide is better established in the literature. Semicarbazide does not interfere either with the chromatogram or with the derivatization reaction of benzaldehyde.

In several tests, it was established that, by acidification with hydrochloric acid instead of sulfuric acid, and a pH range thus set at between 4.0 and 5.1, the derivatization was completed within 45 min and thus produced maximal measurement signals.

The problem described by van Dijk et al. [19] that the excess pressure developed in the reaction vessels by the temperature increase caused the lids to blow off if a hole was not made in the lids, was avoided here by using threaded vessels with a rubber seal (Sarstedt).

### Statistics

Statistical analysis was performed using univariate analysis of variance to test the effect of group and medication status. The analyses were carried out using SPSS statistical software (SPSS, release 10.0.5 for Windows).

## Results

**Figure 1:**
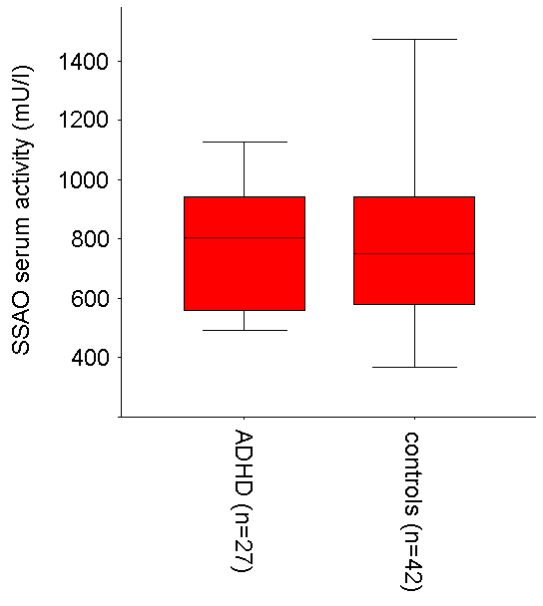
SSAO serum activity (mU/l) in children with ADHD and controls.

In controls and ADHD children the SSAO serum activity (Fig. 1) was within the range of the sample of Boomsma et al. [16] with 147 healthy children and adolescents (about 620 ± 250 mU/l). The somewhat lower SSAO activity in the latter study, compared to the values presented here, might be due to age effects with older children in Boomsma et al. [16].

The univariate analysis of variance revealed no differences between children with ADHD (SSAO activity M = 773, SD = 217 mU/l) and healthy children (SSAO activity M = 775, SD = 256 mU/l) (F = 0.86; p > 0.36; η_p_^2 ^= 0.01; df = 1) and no influence of stimulant medication status (F = 2.52; p > 0.12; η_p_^2 ^= 0.04; df = 1).

## Discussion

The present study investigated the first time extracellularly acting SSAO in children with ADHD to test for its possible role within the neurobiological background of this disorder. No significant differences between children with ADHD and healthy controls in SSAO serum levels were found. Further, stimulant medication status had no influence on the result.

The finding is in line with normal SSAO levels in spontaneously hypertensive rats (SHR; an animal model of ADHD) [20-22] and questions a differentiating influence of SSAO in ADHD.

Reduced quality of measurement underlying the lack of differences could be excluded due to our findings of stable SSAO serum activity in healthy adults (two blood samples per person, three hours apart) [23]. But several limitations might contribute to the absence of found group differences.

In human brain the existence of SSAO is still under consideration [14]. Due to its selective, membrane-bound expression in human brain blood vessels, but not in brain parenchyma (i.e. neurons and glial cells) [12,13] SSAO has been related to the blood-brain barrier in addition to the better known, intracellularly acting MAO [24]. Both metabolize circulating amines to maintain brain homeostasis. The origin of the circulating serum SSAO (analyzed in the present study) and its relationship to the membrane-bound form is still a matter of debate [16]. It seems to be released mainly from vascular endothelial cells [25,26], adipocytes [27] and to some part from skeleton [28]. However at present it is only justifiable to examine serum SSAO, because the examination of the membrane-bound form in humans is restricted to highly invasive techniques of tissue extraction. Thus, the present analysis of SSAO serum activity reflects only one portion of the SSAO system possibly involved in monoaminergic abnormalities in ADHD.

The small effect sizes of aminoxidase differences found in mental disorders, e.g. MAO in schizophrenia [29], might also be present in SSAO and thus contribute to the absence of SSAO differences in our small sample. However, alterations in SSAO activity observed in disease states are generally more dramatic than those reported for MAO [10]. But we detected only small effects according to Cohen [30] concerning the absence of both group differences of SSAO serum activity and of influence of medication status. Nevertheless, there exists some, albeit equivocal, evidence for an ADHD specific alteration concerning catechol-O-methyltransferase (COMT) gene and MAO A and B genes as well as MAO serum levels (e.g. lower levels of platelet MAO activity in untreated children and adolescents with ADHD [3]).

Thus our negative finding concerning group differences of SSAO serum activity has to be interpreted with some caution because it is difficult to be certain to what extent it may reflect a lack of statistical power to detect small effect sizes.

## Conclusion

In sum, there is no evidence for a deviation of SSAO serum activity in ADHD. Hence, extracellularly acting SSAO does not seem to be a promising factor for further research in ADHD. But progress in knowledge of its physiologic role and of the relationship between the membrane-bound and the circulating serum form may open new avenues for research on SSAO in ADHD.

## List of abbreviations

semicarbazide-sensitive amine oxidase (SSAO)

attention deficit hyperactivity disorder (ADHD)

monoamine oxidases (MAO)

high pressure liquid chromatography (HPLC)

electroencephalography (EEG)

## Competing interests

The author(s) declare that they have no competing interests.

## Authors' contributions

VR conceived of the study, participated in its design, recruited half of the patients and drafted the manuscript. HU recruited half of the patients and participated in study coordination. AB performed the statistical analysis. GB carried out the laboratory analyses and helped to draft the manuscript (methods). SB conceived of the study, participated in its design and coordination. AR participated in study coordination and revised the manuscript critically for important intellectual content. All authors read and approved the final manuscript.
